# Effect of Using VR Game-Based Training to Correct Lumbar Curve in Chronic Low Back Pain Patients: Randomized Controlled Trial

**DOI:** 10.3390/healthcare14091207

**Published:** 2026-04-30

**Authors:** Ehab Ahmed, Mohamed Raafat Atteya, Hisham Mohamed Hussein, Rania Youssef, Rehab Ismail, Saud Alrawaili, Enas Abutaleb, Mohamed Eldesoky

**Affiliations:** 1 Department of Public Health, College of Public Health and Health Informatics, University of Ha’il, Hail 55476, Saudi Arabia; em.ahmed@uoh.edu.sa; 2 Department of Physical Therapy, College of Applied Medical Sciences, University of Ha’il, Hail 55476, Saudi Arabia; mr.attya@uoh.edu.sa (M.R.A.); rehab.abutaleb@uoh.edu.sa (R.I.); 3 Department of Basic Sciences for Physical Therapy, Faculty of Physical Therapy, Cairo University, Giza 12613, Egyptmeldesoky@ut.edu.sa (M.E.); 4 Department of Health Management, College of Public Health and Health Informatics, University of Ha’il, Hail 55476, Saudi Arabia; 5 Department of Health and Rehabilitation Sciences, College of Applied Medical Sciences, Prince Sattam Bin Abdulaziz University, Al Kharj 11942, Saudi Arabia; s.alrawaili@psau.edu.sa; 6 Department of Health Rehabilitation Sciences, College of Applied Medical Sciences, University of Tabuk, Tabuk 47512, Saudi Arabia

**Keywords:** chronic pain, hyperlordosis, exercise, TBed, LLA, patient satisfaction

## Abstract

**Background:** Chronic nonspecific low back pain (CNLBP) with lumbar hyperlordosis leads to pain, dysfunction, and poor quality of life. Virtual reality (VR)-based training may enhance exercise engagement and outcomes. This study compared VR-based pelvic rocking training with conventional pelvic rocking training exercises. **Methods:** A triple-blind randomized controlled trial enrolled 100 participants with CNLBP and hyperlordosis, who were randomly allocated into two groups: the group, which performed pelvic rocking exercises using the TBed VR system (TbG), and the conventional group (CG), which performed the same exercises without VR. Both groups completed three sessions per week for eight weeks. Primary outcomes included pain (Numerical Pain Rating Scale, NPRS), lumbar lordotic angle (LLA), lumbar range of motion (ROM), and functional disability (Oswestry Disability Index, ODI). Secondary outcomes were patient satisfaction and commitment to exercise sessions. Assessments were conducted at baseline, immediately post-intervention, and after a one-month follow-up. **Results:** Both groups showed significant improvements in all outcome measures post-treatment (*p* < 0.016). Furthermore, some outcomes—specifically pain, LLA, and extension ROM—continued to improve during the follow-up period. The TbG demonstrated significantly greater reductions in pain, greater ROM improvements, greater functional gains, and higher levels of satisfaction and commitment than the CG (*p* < 0.05). These between-group differences persisted at the one-month follow-up, particularly for pain and ROM, which remained statistically significantly better in the TbG. Moreover, all between-group differences demonstrated medium to high clinical effects (d ≥ 0.3). **Conclusions:** Pelvic rocking exercises using the TBed VR system were superior to conventional exercises in terms of pain, ROM, and function at the immediate and intermediate time points. Using TBed led to better patient satisfaction and higher exercise commitment.

## 1. Introduction

Low back pain (LBP) is widely referred to as the most common musculoskeletal disorder. Nearly everyone is likely to experience at least one episode of LBP during their lifetime [1]. Because it is so prevalent, it is associated with increased work absenteeism, long-term disability, and reduced quality of life. This condition also creates a substantial economic burden, particularly in low-income countries [2].

The causes of LBP can vary widely. In some cases, it begins following an accident or traumatic injury to the lower spine or pelvis. In other circumstances, poor posture or alignment issues, such as anterior pelvic tilt or leg-length discrepancy, may be the main cause. For others, LBP may result from a disc-related problem in the lumbar spine, often accompanied by referred pain into the legs (sciatica). However, in most cases, there is no clear underlying cause, and these cases are classified as nonspecific LBP [3].

Previous studies suggest that the lumbar lordotic angle (LLA) may contribute to LBP [4]. This spinal curve develops during infancy as the child begins to stand upright [5]. LLC helps position the trunk’s center of mass above the hips and offers biomechanical benefits by reducing shear forces on soft tissues and supporting more efficient movement of the lower limbs. However, these advantages may also increase the risk of LBP [4,6].

The LLC value is typically affected by the muscles surrounding the abdomen, lower back, front and back of the hip, and hamstrings [7]. When there is an imbalance in sagittal-plane muscles—such as tight low back and anterior hip muscles combined with weak or overstretched abdominal muscles—an anterior pelvic tilt may occur, leading to an increase in the LLC. In contrast, some patients may exhibit a reduced LLC value due to muscle imbalance [8].

The angle produced by the LLC is called the lumbar LLA. This angle can be accurately assessed with a plain X-ray of the spine and is considered the most accurate method of assessing LLC [9]. LLA can be measured from a lateral X-ray film by determining the angle of intersection between two lines. The first line was drawn along the upper surface of the first lumbar vertebra, and the second line was drawn along the lower surface of the final lumbar vertebral body [10].

Because LBP can arise from many different causes, its treatment approaches also vary. Exercises, manual therapy, physical therapy modalities, as well as medications and surgery, are among the treatment options. The specific characteristics of each patient control the use of each treatment method [11,12].

Pelvic rocking exercises, including anterior and posterior tilts, are a key part of exercise therapy because they increase lower-spine mobility and help rebalance the muscles in the front and back of the body, allowing the lumbar spine to return to its natural curve [13].

TBed is a treatment bed equipped with sensors and divided into four sections. It uses a sensor network to measure pressure from the patient’s body and shows the readings in real time on a connected computer screen. This setup lets patients control different games by using their neck, upper back, and lower back muscles, making it a strong choice for video game–based spinal rehabilitation [14].

While the therapeutic potential of VR for managing CLBP has been increasingly recognized, the specific application of sensorized beds—such as the TBed system—remains markedly under-investigated. Furthermore, prior research has yielded inconclusive findings on the immediate-term impact of VR and has largely overlooked key patient-centric outcomes, including satisfaction and adherence to exercise programs. By focusing on a specific CLBP subpopulation with lumbar hyperlordosis and rigorously evaluating the effects of the TBed system, this trial not only explores a novel therapeutic tool but also adds to the existing knowledge regarding these outcomes. This is particularly important given that low adherence to prescribed exercise—estimated to affect only 40–50% of patients—remains a major barrier in conventional physical therapy [15]. Therefore, the study’s findings on the TBed system’s ability to enhance both satisfaction and adherence offer a vital new perspective on how VR gamification can be leveraged to improve rehabilitation engagement and effectiveness [16,17].

Therefore, this study aimed to examine the effects of incorporating pelvic rocking exercises through VR video gaming on pain intensity, LLA, range of motion (ROM), and function in patients with CNLBP. It also assessed whether adding VR to the exercise program influenced participant satisfaction and commitment.

## 2. Materials and Methods

- Design:

A triple-blind randomized controlled trial.

- Setting:

The study was scheduled to take place from October to December 2025 in a university research laboratory in Ha’il, Saudi Arabia. Ethical approval was granted by the University of Ha’il Ethical Committee (H-2025-675). All procedures adhered to the Declaration of Helsinki [18] and were prospectively registered in clinicaltrials.gov with the code number (NCT06868329). The study adhered to the CONSORT reporting guidelines for randomized clinical trials.

- Duration:

Eight weeks

Assessment timeline: at baseline, after the end of the therapeutic interventions, and after 1 month of the end of the intervention (1-month follow-up).

- Participants:

Participants were enrolled from the university’s local community, including students, staff, and faculty. Recruitment was promoted through e-mail, social media posts, printed notices, and in-person announcements.

The inclusion criteria were males and females aged 18 to 44 years with unilateral, bilateral, or central LBP lasting for 3 months or longer, a pain intensity score of 2 to 6 on the Numeric Pain Rating Scale (NPRS), and evidence of lumbar hyperlordosis, defined as LLA greater than 45° [19], measured using a flexi ruler and Spinal Mouse. The exclusion criteria included acute LBP lasting less than 3 months, specific pain caused by a confirmed pathology such as trauma, surgery, disc lesion, tumor, or adhesions, and LBP associated with sciatica.

- Outcome measures:

A physical therapist with 20 years of experience in orthopedic rehabilitation performed the baseline, immediate post-intervention, and 1-month follow-up assessments. At the initial visit, participants were interviewed to explain the study’s objectives and address any questions. Once informed consent was obtained, additional outcome measures were collected. Participant demographics (gender, age) and anthropometrics (weight, height) were recorded, and BMI was then calculated.

- Primary outcomes:

Intensity of LBP: Pain intensity was assessed using NPRS. This scale consists of a horizontal 10 cm line numbered from 0 to 10. Higher numbers indicate greater pain intensity, while lower numbers indicate lower pain intensity [14]. The validity and reliability of this scale for measuring musculoskeletal pain have been established in previous work [20]. Participants were asked to select the number that represents their current pain level.

Active range of motion of the lumbar spine: Active lumbar flexion and extension range of motion (AROM) were assessed using the Back Range of Motion (BROM) device, which is specifically designed to measure movement in the lumbosacral region. The device consists of two plastic frames—one vertical and one horizontal—each equipped with inclinometers to record sagittal, frontal, and rotational movements of the lower back. The BROM is a valid and reliable tool for evaluating lumbar AROM [21]. In this study, we used the BROM unit (Performance Attainment Associates, Roseville, Minnesota) to measure active lumbar flexion and extension according to previously described methods [22]. AROM was calculated by subtracting the initial position angle from the final position angle reached during flexion or extension.

Lumbar lordotic angle: The LLA can be determined on a lateral lumbar X-ray by drawing one line along the superior endplate of L1 and another along the inferior endplate of L5, then measuring the angle at their intersection. An alternative method employs a 60 cm flexicurve ruler—a plastic-coated steel strip that conforms to the natural curve of the lumbar spine and retains that shape- can be used to assess LAA. The flexicurve ruler is considered non-invasive, simple to apply, and easy to use in clinical settings, so we adopted this method in the current study. The reliability and validity of the flexicurve method have been demonstrated [23]. The assessor, who was blind to each patient’s intervention group, measured lumbar lordosis with patients standing upright, knees extended, and feet about shoulder-width apart. First, the bilateral posterior superior iliac spines are palpated, and their midpoint is marked as S2. To locate L1, the examiner finds the highest point of the iliac crest (corresponding to L5), then counts upward to L1. With the patient facing forward, a flexible ruler is gently pressed along the line between L1 and S2 until it conforms to the lumbar curve. That contour is then traced onto paper, and the angle Ө is calculated using: Ө = 4 × arctan (2 H/L), where L is the straight-line distance between the curve’s endpoints, and H is the maximal perpendicular height from L to the curve [24]. A normal LLA is around 30°, while values above 40° indicate hyperlordosis [25].

Function: The Oswestry Disability Index (ODI) was employed to quantify functional impairment. It comprises ten sections that address back-related pain and its impact on everyday activities. Each section offers six response options scored from 0 to 5, where the first choice equals 0 points, the second equals 1 point, and so on. Summing all item scores yields a raw total between 0 and 50, which can also be expressed as a percentage; in both formats, lower scores denote better function and higher scores indicate greater dysfunction. In this study, we used the raw scores for analysis because they are more sensitive to small changes than percentage values [26]. Previous research has demonstrated the ODI’s strong validity and reliability, with intraclass correlation coefficients ranging from 0.88 to 0.96 [27].

- Secondary outcomes:

Satisfaction level: Satisfaction with the physical therapy program was assessed using a 0–10 numerical rating scale adapted from the NPRS, where 0 indicated no satisfaction and 10 the highest possible satisfaction [28]. However, the psychometric properties of this scale are not well established in the literature; several studies have used it to assess patient satisfaction and experience across different healthcare contexts [29,30]. Patients were asked, “How would you rate your satisfaction with the introduced physical therapy program?” and selected a number from 0 (not at all satisfied) to 10 (completely satisfied) to indicate their current level of satisfaction [14].

Commitment to exercise sessions: This outcome was calculated by dividing the total number of successful sessions per group (the number of sessions conducted successfully in each group) by the total number of sessions (1200 per group), then multiplying the result by 100 to obtain a percentage of success for each group [14].

### 2.1. Interventions

The intervention program in this study was designed to consist of 24 sessions (applied 3 times per week) for eight weeks. Each session lasted an average of 40 min.

#### 2.1.1. Pelvic Rocking Exercises Using TBed

The intervention involved pelvic rocking exercises involving the lower and upper back and lower and upper abdominal muscles [31]. using the TBed system (software name Postural Suite 1.2.1, Techno body©, Roma, Italy) paired with a VR video-gaming interface. Patients in the TbG were instructed to lie supine on the TBed and contract the low back muscle groups to press downward on the TBed surface. This pressure engaged internal sensors, which, in the game, triggered a virtual gun to shoot at flying fruits. The game offered three difficulty settings—easy, medium, and hard—and included two task types: posterior pelvic tilts to engage the lower back and scapular presses to engage the upper back. Each session began with one familiarization trial, followed by three recorded game trials for the lower back and three for the upper back. Participants started at the easy level and advanced to higher levels once they demonstrated mastery and achieved high scores. Initially, all gaming took place in a supine position; beginning in week three, sessions were conducted in a reclined posture. Patients in the TbG group only received this type of exercise.

#### 2.1.2. Pelvic Rocking Exercises Using the Conventional Technique

Patients were asked to assume a supine position with both knees semi-flexed. A small pillow was placed under the lower back, and another under the flexed knees for comfort. The patient was asked to tuck the abdominal muscles inward, press the lower back into the underlying pillow, hold for 6 s, then relax. After a few seconds of rest, the patient should repeat this procedure for 3 sets × 10 repetitions per session during the first 2 weeks. The number of sets was increased based on each patient’s adaptation and fatigue threshold. Three minutes of rest were allowed between every set. Patients in the control group only received this type of exercise.

#### 2.1.3. Moist Heat

A 20 min automatic moist hot pack (Besmed© BE-267, Taipei, Taiwan) of suitable size was used to apply superficial heat. The heat level was set to medium. A moist environment was ensured by placing a wet sponge between the pack and its cotton outer cover. The duration was calculated after 8 min of warming up (when the temperature reached its maximum). The patient-reported sensation should be moderate warmth. A thermal sensation test using two test tubes was performed before applying heat therapy to avoid any adverse effects [32]. This intervention was applied to patients in both groups.

#### 2.1.4. Hamstring and Back Muscles Stretching

Manual passive stretching of the hamstring and lower back muscles was performed in three 30-s repetitions per session. Participants adopted a long-sitting posture with knees fully extended and feet together. They were instructed to hinge at the hips and lean forward as far as possible toward their feet. Once this end-range was reached, the therapist applied overpressure by placing hands on the participant’s upper back and gently pushing forward [33]. This intervention was applied to patients in both groups.

### 2.2. Sampling Size Calculation

The GPower (version 3.1.9.7) software was used to conduct sample size calculation. ANOVA with Repeated measures and a within–between interaction category was used. The data used were a medium effect size on the ODI scale (0.30), alpha level 0.05, power 80%, number of groups 2, a correlation between repeated measures of 0.2, and a sphericity correction value of 0.5. This calculation yielded 42 patients per group. To anticipate potential drop-out, 50 patients were enrolled in each group.

### 2.3. Allocation, Concealment, and Blinding

To ensure adequate allocation concealment, a computer-generated randomization sequence was created, and a permuted block design with block sizes of 4 and 6 was used to maintain a 1:1 allocation ratio. Allocation concealment was conducted by a coworker who was not involved in assessment or treatment. The therapist and assessor were not involved in the allocation process. After the allocation, the coworker assigned each patient a unique code number to use throughout the study. After the start of the study, only the therapist was allowed to uncover the allocation sequence codes to provide the appropriate treatment for each patient group. The assessors and patients remained blinded throughout the study. Additionally, to ensure the statistician remained blinded, the data from the two groups were labeled using randomly assigned English letters.

### 2.4. Statistical Design

All statistical analyses were performed using SPSS version 23 (Chicago, IL, USA). Data are presented as mean ± standard deviation. Normality was assessed with the Kolmogorov–Smirnov test. Between-group differences were analyzed using one-way ANOVA, while within-group differences were examined using repeated-measures ANOVA. A two-sided *p* < 0.05 was considered statistically significant. Tukey’s post hoc test was used when needed. Effect sizes were estimated using Cohen’s *d*. The study did not use an intention-to-treat analysis, as we increased the sample to account for drop-out.

## 3. Results

**Figure 1:**
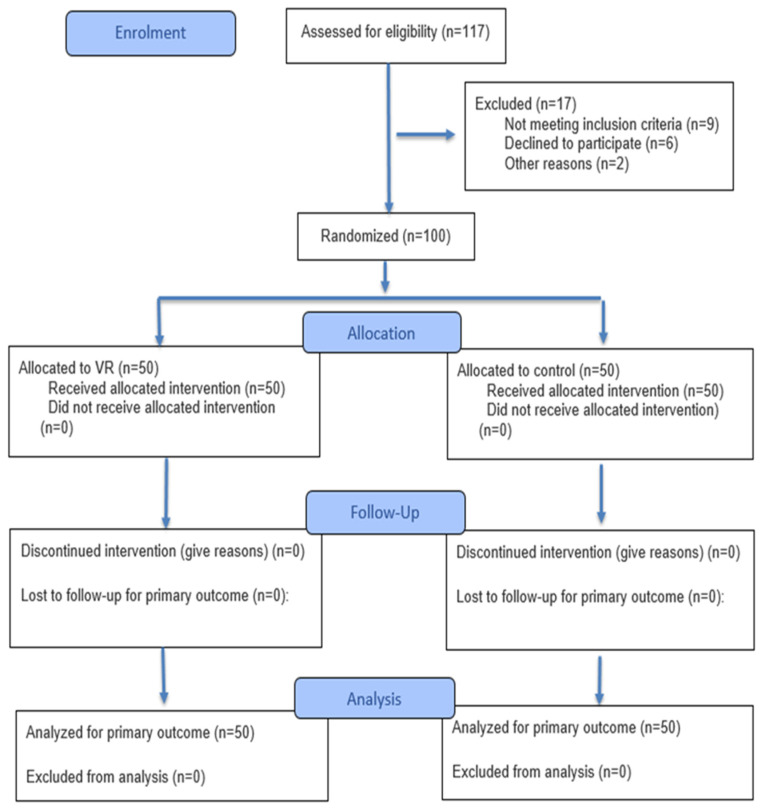
Flow Diagram.

This study included 117 male participants, of whom only 100 met the criteria for LBP and an exaggerated lumbar curve. These participants agreed to participate in the study, as shown in Figure 1. Recruitment started in October 2025 and continued until the required number was reached, and the study was completed with follow-up in December 2025. Participants were allocated to two equal groups, and outcomes were measured at three time points (pre-, immediately post-program, and 1 month after the program for follow-up). None of the cases showed adverse effects, as the program followed safety guidelines. Age, weight, height, and body mass index (BMI) were measured and compared between groups (Table 1).

### 3.1. Within-Group One-Way ANOVA Results

The within-groups analysis of the TbG demonstrated statistically significant difference in pain (F = 98.67, DF = 3, *p* < 0.001), LLA (F = 18.67, DF = 3, *p* < 0.001), flexion ROM (F = 138.22, DF = 3, *p* < 0.001), extension ROM (F = 136.31, DF = 3, *p* < 0.001), and function (F = 17.15, DF = 3, *p* < 0.001). Similarly, the CG demonstrated statistically significant differences in pain (F = 18.69, DF = 3, *p* < 0.001), LLA (F = 14.34, DF = 3, *p* < 0.001), flexion ROM (F = 94.11, DF = 3, *p* < 0.001), extension ROM (F = 204.70, DF = 3, *p* < 0.001), and function (F = 41.13, DF = 3, *p* < 0.001) (Table 2).

### 3.2. The Post Hoc Analysis for All Outcomes

All outcome measures in both groups demonstrated statistically significant differences (*p* ≤ 0.016) between baseline and post-treatment, and baseline and follow-up. These findings were also evident clinically by medium to high effect size (*d* < 0.30). Flexion AROM in the TbG and function in the CG were the only outcomes that demonstrated a statistically significant difference and medium effect size at follow-up compared to post-treatment (*p* = 0.003, *d* = 0.601) and (*p* < 0.001, *d* = 0.534), respectively (Table 2).

### 3.3. Between-Groups Analysis

There were statistically significant differences in pain scores between groups post-treatment and at follow-up (*p* < 0.001) with a medium effect size. Regarding ROM, statistically significant differences were observed only at follow-up, with a low-to-medium effect size (Table 3). There were statistically significant differences in function between groups in both post-treatment and follow-up assessments, with a medium effect size. There was a statistically significant difference between groups in satisfaction level (*p* < 0.001, *d* = 1.09).

Regarding participants’ commitment to therapy, 1096 sessions out of 1200 (91%) were successfully conducted in the CG, while in the TbG, 1196 sessions were conducted out of 1200, which represents a success rate of 99.6%.

## 4. Discussion

This study compared the effects of regular pelvic rocking exercise with those of VR-augmented exercise in patients with LBP and hyperlordosis. The findings demonstrated that both techniques were equally effective in favor of LLA and AROM. However, VR-augmented exercises using TBed demonstrated better outcomes in pain, function, satisfaction, and commitment to exercise sessions than regular exercise.

Previous literature contains several attempts to merge VR technology as a new trend in the rehabilitation of LBP and related dysfunctions. However, the findings were inconclusive [34]. As the current study did, previous literature [16,17,34] focused on outcomes such as pain and function. However, the current study added several additional outcomes, including the value of the LLA, AROM of the lumbar spine, patient satisfaction, and level of commitment to exercise sessions.

The findings regarding pain and function reported in the current study agreed with those reported in previous studies. Afzal et al. compared routine physical therapy alone with routine physical therapy plus VR exercises delivered through a non-immersive kinetic exergame system (Model V.2) displayed on an LCD screen. Although both groups improved, the VR group achieved significantly greater benefits in favor of pain and function. These findings support current study findings [17].

On the other hand, Eccleston et al. found no significant between-group differences in pain intensity or function either after treatment or at follow-up. This difference from our results may be related to variations in intervention emphasis, VR technology, and participant characteristics. Specifically, their approach was more psychological and embodiment-based, whereas ours focused on biomechanical correction through exercise. They used an Oculus Quest headset, while our study used the TBed sensorized system, and their participants represented a broader CLBP population with fear-avoidance tendencies rather than a subgroup with hyperlordosis [16].

Groenveld et al. compared a VR-based behavioral therapy application (Reducet) on an Oculus Go head-mounted, (Facebook Technologies, LLC, Menlo Park, CA, USA) display with standard care. Their findings partially align with ours, but with notable differences. They observed a significant treatment effect for the daily worst pain score and the least pain score. Importantly, they reported a reduction in analgesic consumption in the VR group. On the other hand, unlike the current study, Groenveld et al. found no significant improvement in function, which could be attributed to: (1) shorter intervention duration in Groenveld’s study (4 weeks vs. our 8 weeks); (2) lower total VR exposure (approximately 5 h total vs. our 18 supervised sessions) [15].

Kim et al. investigated simulated horseback riding for CLBP using a mechanical riding simulator rather than VR, so direct comparison with our VR-based intervention is limited. Nevertheless, their results, showing significant improvements in pain and function, support our conclusion that VR-assisted exercise programs can be effective in managing CLBP [7].

The differences in findings between our study and some previous work may be due to differences in the exercises used. For example, Afzal et al. added VR to routine physical therapy and compared its effects with those of routine physical therapy alone [31]. In the study conducted by Li et al. [35]. The VR group received magnetic therapy alongside VR training, while the other group performed an abdominal drawing-in maneuver under real-time ultrasound and four-point kneeling exercises. Additionally, the wide variations of the VR technologies and tools used in previous work could be another contributing factor. In Afzal’s study [17], a Kinetic exergames device was used, whereas in Eccleston’s study, an Oculus Quest and Touch VR headset and a handheld controller were used [16]. Meanwhile, Groenveld’s study used an Oculus Go head-mounted display [15]. Many other variations of VR were also used in other related literature [34].

Unfortunately, the literature was scarce regarding LLA, ROM, patient satisfaction, and commitment to exercise sessions, hindering an appropriate discussion of these important outcomes. Satisfaction and commitment to exercise session outcome measures should be incorporated into VR-based studies to highlight the possible recreational and motivational influences of VR on the patient and the rehabilitation program. Also, these outcomes could direct our attention to the type and mode of VR that meet the patient’s expectations, achieve the highest level of motivation and, consequently, commitment to exercise sessions, and result in better rehabilitation. However, a more robust assessment using accurate assessment tools, especially for satisfaction and commitment, could be incorporated in future work.

This study is limited to those patients with LBP and hyperlordosis; the results cannot be generalized to the wider LBP population, so the practical use of the current findings should be taken with caution. The one-month follow-up period may not be enough to report such a chronic problem and its prolonged course. So, future studies could implement longer follow-up periods. The results of this study should be interpreted with caution because the statistical design did not implement intention-to-treat analysis, and the numerical scale used for assess patient satisfaction lacks appropriate validation evidence. The relatively low sample size might limit the generalizability of the findings, especially to other LBP populations.

## 5. Conclusions

VR-augmented pelvic rocking exercises using the TBed system may represent a promising technique for managing chronic low back pain with hyperlordosis. The findings suggest potential for better outcomes in pain, functional outcomes, patient satisfaction, and exercise commitment, with benefits possibly persisting for at least 1 month after the intervention.

## Figures and Tables

**Table 1:** Comparisons of the characteristics of the TbG and control groups.

	TbG (n = 50)	CG (n = 50)	F	*p*
Age (Y)	27.96 ± 7.10	27.40 ± 7.28	0.151	0.698
Weight (kg)	68.06 ± 7.55	67.06 ± 7.65	0.432	0.512
Height (m)	1.70 ± 0.08	1.69 ± 0.09	0.449	0.504
BMI (w/h^2^)	27.50 ± 7.97	27.61 ± 5.05	0.011	0.917

TbG, Tbed group; CG, control group; n, number of participants in the group; F, ANOVA; *p*, significance; Y, year; kg, kilogram; m, meter; w/h^2^, weight divided by squared height.

**Table 2:** Within-group comparisons at baseline, post-treatment, and follow-up.

	Group	Values	Pre vs. Post Intervention m ± SD		Pre vs. Follow-Up m ± SD		Post vs. Follow-Up m ± SD	
Pain * (NPRS)	TbG	m ± SD	4.04 ± 1.21	1.96 ± 0.90	4.04 ± 1.21	1.82 ± 0.82	1.96 ± 0.90	1.82 ± 0.82
		*p*	<0.001		<0.001		0.054	
		Cohen’s *d*	1.96		2.17		0.164	
	CG	m ± SD	4.10 ± 1.32	2.86 ± 1.44	4.10 ± 1.32	2.56 ± 1.24	2.86 ± 1.44	2.56 ± 1.24
		*p*	<0.001		<0.001		0.854	
		Cohen’s *d*	0.917		1.23		0.230	
Lordotic angle *	TbG	m ± SD	51.20 ± 5.10	47.68 ± 5.77	51.20 ± 5.10	48.86 ± 5.34	47.68 ± 5.77	48.86 ± 5.34
		*p*	<0.001		<0.001		0.137	
		Cohen’s *d*	0.646		0.448		0.212	
	CG	m ± SD	50.90 ± 5.54	49.44 ± 5.63	50.90 ± 5.54	49.02 ± 5.18	49.44 ± 5.63	49.02 ± 5.18
		*p*	<0.001		<0.001		0.779	
		Cohen’s *d*	0.264		0.355		0.077	
AROM * (flexion)	TbG	m ± SD	37.28 ± 7.00	48.82 ± 5.55	37.28 ± 7.00	49.86 ± 4.94	48.82 ± 5.55	49.86 ± 4.94
		*p*	<0.001		<0.001		0.227	
		Cohen’s *d*	1.82		2.07		0.197	
	CG	m ± SD	38.16 ± 7.24	46.49 ± 6.82	38.16 ± 7.24	47.22 ± 6.34	46.49 ± 6.82	47.22 ± 6.34
		*p*	<0.001		<0.001		0.997	
		Cohen’s *d*	1.18		1.33		0.110	
AROM * (Extension)	TbG	m ± SD	14.50 ± 2.93	20.32 ± 2.92	14.50 ± 2.93	21.88 ± 2.22	20.32 ± 2.92	21.88 ± 2.22
		*p*	<0.001		<0.001		0.003	
		Cohen’s *d*	1.89		2.83		0.601	
	CG	m ± SD	13.40 ± 3.01	20.12 ± 3.14	13.40 ± 3.01	20.44 ± 2.67	20.12 ± 3.14	20.44 ± 2.67
		*p*	<0.001		<0.001		0.718	
		Cohen’s *d*	2.15		2.43		0.109	
Function * (ODI)	TbG	m ± SD	23.30 ± 5.26	17.96 ± 3.62	23.30 ± 5.26	22.32 ± 6.12	17.96 ± 3.62	22.32 ± 6.12
		*p*	<0.001		0.994		<0.001	
		Cohen’s *d*	1.18		0.171		0.867	
	CG	m ± SD	23.00 ± 5.64	21.28 ± 4.89	23.00 ± 5.64	18.44 ± 5.70	21.28 ± 4.89	18.44 ± 5.70
		*p*	<0.001		<0.001		<0.001	
		Cohen’s *d*	0.325		0.804		0.534	

NPRS, numeric pain rating scale; AROM, active range of motion; ODI, Oswestry disability index; *, primary outcome; TbG, TBed group; CG, control group; m, mean; SD, standard deviation. Significant when *p*-values ≤ 0.016.

**Table 3:** Between-group analysis at baseline, post-treatment, and follow-up.

	Time of Assessment	TbG (n = 50)	CG (n = 50)	MD	*p*	*d*
		m ± SD	m ± SD			
Pain * (NPRS)	Baseline	4.04 ± 1.21	4.10 ± 1.32	0.06	0.814	0.047
	Post treatment	1.96 ± 0.90	2.86 ± 1.44	0.90	<0.001	0.749
	Follow-up	1.82 ± 0.82	2.56 ± 1.24	0.47	<0.001	0.703
Lordotic angle *	Baseline	51.20 ± 5.10	50.90 ± 5.54	0.30	0.779	0.056
	Post treatment	47.68 ± 5.77	49.44 ± 5.63	1.76	0.126	0.308
	Follow-up	48.86 ± 5.34	49.02 ± 5.18	0.16	0.880	0.030
AROM * (flexion)	Baseline	37.28 ± 7.00	38.16 ± 7.24	0.88	0.538	0.123
	Post treatment	48.82 ± 5.55	46.49 ± 6.82	2.33	0.134	0.374
	Follow-up	49.86 ± 4.94	47.22 ± 6.34	2.64	0.022	0.464
AROM * (Extension)	Baseline	14.50 ± 2.93	13.40 ± 3.01	1.10	0.067	0.370
	Post treatment	20.32 ± 2.92	20.12 ± 3.14	0.20	0.743	0.065
	Follow-up	21.88 ± 2.22	20.44 ± 2.67	1.44	0.004	0.586
Function * (ODI)	Baseline	23.30 ± 5.26	23.00 ± 5.64	0.30	0.784	0.055
	Post treatment	17.96 ± 3.62	21.28 ± 4.89	3.32	<0.001	0.771
	Follow-up	22.32 ± 6.12	18.44 ± 5.70	3.88	0.001	0.656
Satisfaction ^¥^	Post treatment	6.06 ± 0.91	4.72 ± 1.47	1.34	<0.001	1.09

NPRS, numeric pain rating scale; AROM, active range of motion; ODI, Oswestry disability index; *, primary outcome; ^¥^, Secondary outcome; TbG, TBed group; CG, control group; m, mean; SD, standard deviation; MD, mean difference; *p*, significance level; *d*, effect size.

## Data Availability

The data presented in this study are available on request from the corresponding author.
